# On the validity and consistency of misjudgment of stepping ability in young and older adults

**DOI:** 10.1371/journal.pone.0190088

**Published:** 2017-12-21

**Authors:** Nick Kluft, Sjoerd M. Bruijn, Roel H. A. Weijer, Jaap H. van Dieën, Mirjam Pijnappels

**Affiliations:** Department of Human Movement Sciences, Vrije Universiteit Amsterdam, Amsterdam Movement Sciences, Amsterdam, The Netherlands; Purdue University, UNITED STATES

## Abstract

Disparities between perceived and actual physical abilities have been shown in older adults and may lead to balance loss or falls. However, it is unclear whether one’s misjudgment is an inherent trait and thus consistent across different tasks, and whether this misjudgment is age-related. We measured the degree of misjudgment in young and older adults on four different stepping tasks; stepping over a raised bar, crossing a declining cord by stepping over it at a self-selected height, crossing a virtual river by stepping over it at a self-selected width, and making a recovery step after release from an inclined position. Before comparison, we carefully checked the validity of the different tasks to determine the misjudgment. No substantial differences were found in the amplitude of the misjudgment between the age groups, and the degree of misjudgment did not transfer across different stepping tasks. However, since only one task (i.e., stepping over a raised bar) met our criteria for validly assessing one’s misjudgment, it remains unclear whether the degree of misjudgment is task-specific or an inherent trait. These findings stress the importance of testing the construct validity of the task, prior to the examination of the misjudgment of stepping ability.

## Introduction

Motor actions allow humans to interact with the environment, however, there is a rich variety of movements that can fulfill the same motor task [1]. The selection process, or planning of a motor task is crucial for success of the action [2]. Recent studies showed that the selection process depends on one’s perceived ability to perform the intended action [3–5]. This entails that one’s ability must be judged prior to the selection process. Besides judgment of the self, adequate perception of the task at hand is required for successful execution of the task. Healthy young adults can cope with small errors in this judgment, but for older and more fragile adults, an inadequate selection could have large consequences (e.g., causing a fall). Furthermore, ageing is accompanied by physical and cognitive decline [6, 7], a reduction in processing speed [8–10], and neural plasticity [11], which could all be facets that contribute to the introduction of errors in making perceptual judgments that suit one’s capability.

While falls in older adults are associated with age-related physical decline, it is suggested that accidental falls in older adults might also be associated with the misjudgment of actual physical abilities [12, 13]. It could therefore be useful to incorporate the misjudgment in existing fall prediction models, to improve the predictive power of these models. However, to be applicable in fall prediction models, adding misjudgment (i.e., a combination of either or both an over-or underestimation of one’s own motor capacities and the misperception of the environmental or task constraints) is beneficial only if this is an inherent trait and thus consistently observed across different motor tasks.

Consistency in risk-taking behaviour, which could be considered a concept analogue to misjudgment, was studied by O’Brien and Ahmed [14, 15]. Subjects that overestimate their ability might be inclined to accept higher risk than under-estimators [12], who in turn, are more likely to bypass activities to avoid risk exposure out of fear of falling [16]. O’Brien and Ahmed found young individuals to behave consistently as either risk-seeking or risk-adverse across tasks when moving a cursor in a vertical environment as close to the edge of a cliff as possible without moving beyond the edge, either by arm movements or by whole-body leaning movements. However, since the target and the cliff were displayed on a computer screen and the target was controlled via a robotic device, an extra layer of visuomotor control was introduced. It remains unclear whether a similar consistency in the degree of misjudgment holds for real-world motor tasks that require balance control, such as stepping.

Misjudgment has been directly quantified in older adults in stepping accuracy [17] and stepping over a raised bar [18, 19]. Although these studies found different degrees of misjudgment in their tasks, they did not compare with other tasks, nor did they assess the construct validity of the task in question, which complicates comparison between studies.

**Fig 1:**
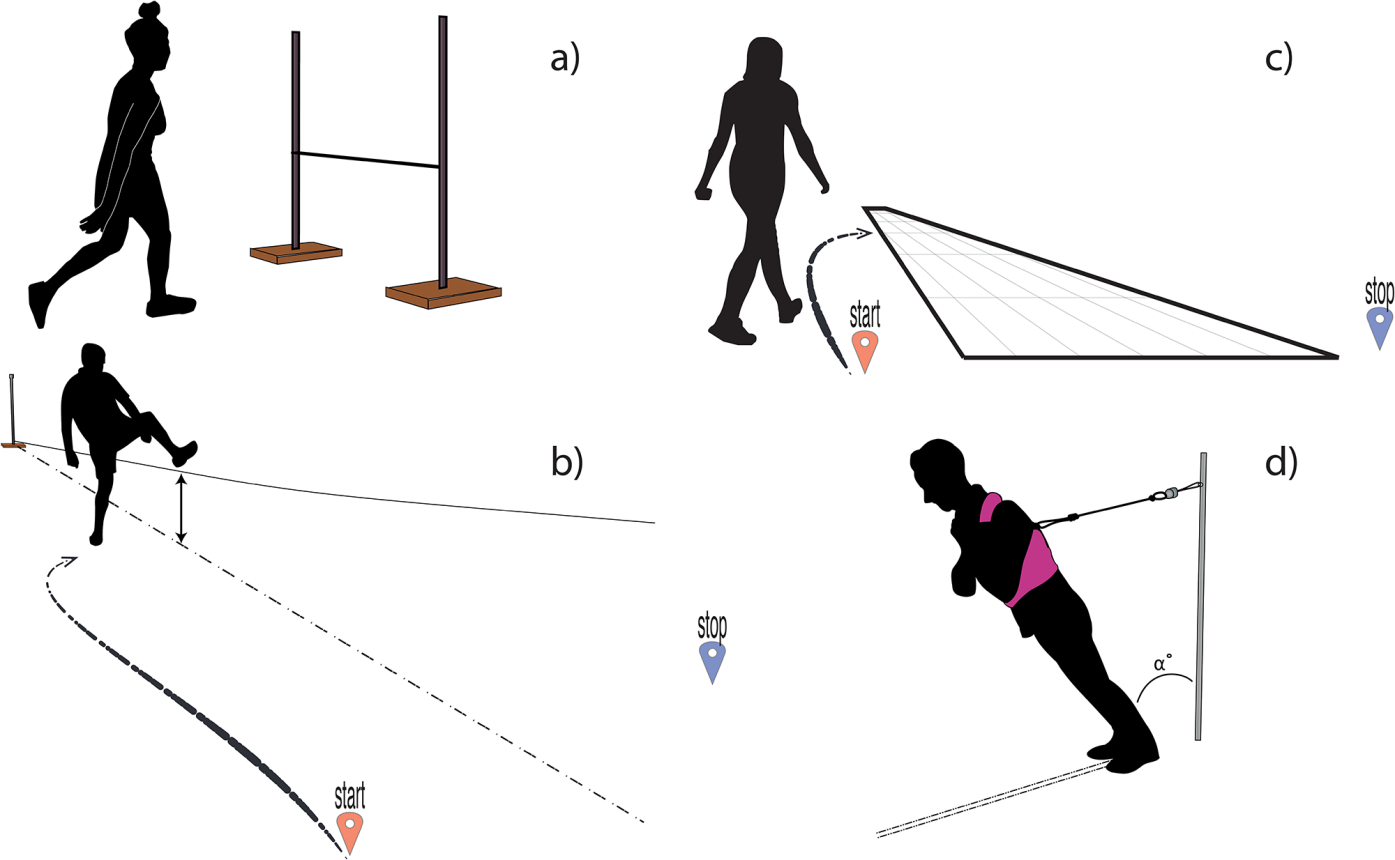
Visualisation of the four motor tasks. **(a)** Stepping over a raised bar (‘bar’). **(b)** Crossing a declining cord by stepping over it at a self-selected height (‘cord’). **(c)** Crossing a virtual river by stepping over a peace of paper at a self-selected width (‘river’). **(d)** Recovery from a forward fall after an unexpected release from an inclined position (‘recovery’). Leaning angle is depicted by the α.

Our overall aim was to unravel whether misjudgment is an inherent trait that transfers to other stepping tasks in young and older adults, thereby advancing this novel framework to help explain falls in older adults. To do so, we set criteria to assess the construct validity of the tasks addressing one’s (mis)judgment of physical ability. We focused on stepping ability, since stepping does initiate locomotion by moving the center of mass outside the base of support, and it is an important strategy to regain balance after a perturbation [20, 21]. Participants judged their physical abilities in four different stepping tasks; stepping over a raised bar, crossing a declining cord by stepping over it at a self-selected height, crossing a virtual river by stepping over it at a self-selected width, and making a recovery step after release from an inclined position (Fig 1). First the construct validity of each of the four tasks was evaluated. When tasks properly assessed physical ability as well as perceived ability, we expected correlations between both these aspects across tasks. Subsequently, we hypothesised that misjudgment is an inherent trait and therefore consistent across different stepping tasks within individuals. As older adults are more likely to misjudge their abilities because of age-related changes, we measured a group of young adults too, in order to establish the validity of the tasks. Any differences in how the perceived ability relates to the actual ability between older and young adults, is an argument against the validity of a task. After determining the validity of the tasks, we explored to what extent misjudgment is affected by ageing, by comparing the degree of misjudgment of young and older adults.

## Materials and methods

### Participants

Fifteen healthy older adults (mean age 74, SD 5.3 and range [67–83] years, 11 females) and 9 healthy young adults (mean age 24, SD 1.5 and range [22–27] years, 5 females) participated in this study (see Table 1 for a detailed participant description). We excluded subjects who had any self-reported musculoskeletal or neurological disorders, major trauma in the last year, mini mental state examination score of 24 or lower, or who took medication which could have affected their gait stability. All participants could walk continuously for at least 10 minutes without any assistive walking device. The protocol was approved by the local research ethics committee (VCWE-2016-077). Participants were recruited from June to August 2016 through flyers, which were spread in popular facilities for older adults in Amsterdam, such as community centers and bridge clubs. When interested, participants were explained the aim and procedures of the experiment by telephone and an appointment was set for the measurements. The younger adults were university students who were personally contacted by one of the experimenters. Prior to the measurements, participants were again informed about the experimental procedures and signed an informed consent.

**Table 1:** Descriptive statistics of participants in both age groups. The mean values (M) and standard deviation (SD) is given for the different descriptives. Difference (t-statistics) between the two age groups are shown (*:p<0.05, **:p<0.01, ***:p<0.001).

Descriptives:				
	Young adults	Older adults	Entity	Statistic
Gender	11/4	5/4	female/male	
Age	24 ± 1.5	74 ± 5.28	years	
Weight	69 ± 13.0	74 ± 14.2	(M±SD) kg	*t* = 0.84
Height	171 ± 9.4	167.6 ± 8.9	(M±SD) m⋅10^−2^	*t* = -0.93
MMSE	n/a	28.5 ± 1.6	(M±SD) points	
FES-I	n/a	20.8 ± 5.3	(M±SD) points	
ABC	n/a	81.5 ± 16.0	(M±SD) %	
Grip strength	36.7 ± 11.4	26.3 ± 7.8	(M±SD) kg	*t* = -2.66*
TMT				
part A	20.6 ± 3.7	36.1 ± 12.0	(M±SD) seconds	*t* = 3.74***
part B	43.0 ± 19.5	82.7 ± 35.5	(M±SD) seconds	*t* = 3.08**
part B—part A	22.4 ± 20.4	46.6 ± 28.7	(M±SD) seconds	*t* = 2.21*

MMSE: mini mental state examination, FES-I: Falls Efficacy Scale International, ABC: Activities-specific Balance Confidence scale, TMT: Trail Making Test, n/a: Not Available

### Protocol

In the first part of the experiment, we measured grip strength (A5401 Digital Hand Grip Strength Dynamometer, Take, Niigata, Japan), trail making test (TMT, [22]), leg length, body height, and body weight. The Falls Efficacy Scale International (FES-I, [23, 24]) and the Activities-specific Balance Scale (ABC, [25]) were also administered in the older adult group.

In the second part of the experiment, participants executed four tests (Fig 1) aimed at quantifying first their perceived and second their actual physical abilities. For the first task in the test battery (‘bar’), we asked participants what they believed the maximum bar height would be at which they could still step over a raised bar (Fig 1a). A bar (2 cm x 2 cm x 137 cm) was attached to two stands using magnets, so the height of the bar was easy to adjust and a light touch caused the bar to fall. For the perceived ability on this task, the participant stood 2 metres in front of the bar stands. The experimenter slowly moved the bar either upwards or downwards in four consecutive trials, and in each trial, the participant had to say stop at their selected height. The participant was given the opportunity to adjust this height when they felt it was not at their indicated height. The mean bar height of four repetitions served as the perceived physical ability measure. For their actual ability on this task, we tested the actual reachable bar height (these procedures were adapted from [18]). Participants started at a bar height of 5 cm, then we increased the bar height by 10 or 5 cm based on the ease with which participants could step over the bar. The bar was attached to two stands using magnets, so a light touch caused the bar to fall. When the height was reached at which the bar was knocked off we asked to try this height again. When the participants failed a second time, we lowered the bar by 2.5 cm and instructed the participants to try again. The highest successfully achieved height was recorded as the actual bar height.

For the second task (‘cord’), we placed the two stands twelve meters apart and placed a string diagonally between them; at the one end, the string was raised 1.2 meters above the ground (high string stand) and at the other was at ground level (Fig 1b). For the perceived ability of this task, participants were standing at the starting point (next to the high string stand) and were instructed to get to the stopping point at the other side of the high string stand as quickly as possible. This task required walking to a lower part to the string, crossing the cord at the height of their own choosing, with a trade-off between the time needed for walking to a lower cord level, versus the ease with which the line could be crossed. This trade-off should drive the participants to cross the cord at about the height of their perceived maximum ability. The chosen location served as a measure of the perceived ability. For the actual ability of this task, we used the maximal bar height at the ‘bar’ task at which participants could step over.

In the third task (‘river’), we instructed participants to walk along a virtual river, i.e., twelve meter long and tapered piece of paper, and to step across it at a location of their own choice (Fig 1c). Again, participants started at the widest end of the river and were instructed to walk down the river and cross it to return to the widest part of the river as quickly as possible. The position where participants crossed was recorded and served as measure of the perceived maximum step length. The actual maximum step length was determined by stepping inside a rectangular target. In a similar fashion to the actual maximum ‘river’ height, the distance to the target was increased until participants failed to step inside the target twice.

In the final task (‘recovery’), the ability to recover from an impending forward fall by stepping was evaluated [26, 27]. Participants wore a safety harness that was secured with ropes to the ceiling. A force transducer was inserted between the ceiling and the harness, enabling fall detection based on the harness’ support. Participants were instructed to keep their body straight and their arms crossed to their chest while they leant forward, supported by a rope attached to the wall behind them (Fig 1d). The leaning angle (i.e., the angle between participant and the wall) was increased, and for each angle, participants indicated whether they believed they could still recover with only one step if the rope was to be released. After this procedure, the largest angle they could actually recover from was determined. The first angle was always set at 5 degrees so all participants could recover themselves. Then, we increased the angle until participants could not recover, again using the same protocol as described above for the actual reachable height and actual maximum step length. Harness support above twenty percent of the participant’s body weight was classified as an unsuccessful trial [28].

### Statistical analysis

First, we tested for possible differences in perceived and actual physical abilities between young and older adults, using independent samples permutation t-tests, in which p-values were adjusted using the max-statistic method [29]. We used this permutation based approach rather than a conventional t-test with, for instance, Bonferroni adjustment, to correct for the multitude of variables that we analysed.

Next, construct validity of the tasks in the test battery was tested using the following criteria: 1) the perceived and actual physical ability measure of one task should relate highly to the same measures of another task, 2) the relation between perceived and actual physical ability should be linear.

The first criterion guarantees that the perceived and actual measures are representative of subjects’ perceived and actual physical abilities; the second criterion ensures that for that task the subjects’ perceived ability is indeed positively and linearly related to their actual physical ability, albeit that an offset from the identity line may exist. If subjects’ perceived ability is not linearly related to their actual physical ability, it could be that they simply cannot make a valid estimate of what they can do. Ideally, perceived and actual ability would cluster around the identity line, and we have previously used the distance from the identity line as a measure of misjudgment [17]. However, offsets with respect to the identity line may exist, for instance, due to the risk involved in making errors [14]. While this offset would not be a problem when assessing misjudgment using only one task, it may lead to problems when trying to compare misjudgment measures between different tasks, as they may have different offsets (even in different units). To examine the consistency of the actual abilities, and perceived abilities across tasks (i.e., test for the first criterion), a permutation test based on Pearson’s correlation coefficient was used. To control for the family wise error rate, p-values were adjusted using the max-statistic method [30].

For verification of the second criterion, the linearity of the relation between the perceived and actual ability was assessed by comparing the small sample-size corrected Akaike information criteria (AIC) of a linear model with an alternative second order quadratic model [31]. The difference between the AIC of the linear and the alternative model (Δ AIC) was calculated by subtracting the AIC of the alternative model from the linear model (Δ AIC = AIC_alternative_ - AIC_linear_); with positive values indicating a better fit of the linear model in terms of the tradeoff between the model’s complexity and accuracy. Finally, to quantify misjudgment and test for its consistency across tasks, the association between perceived and actual ability was determined using a linear regression model. To determine possible differences between the slopes of the linear fit of the two age groups, an interaction term was added to the linear regression. Any substantial differences in the regression coefficients between the age groups would affect the comparison of the degree of misjudgment between groups and between tasks. For those tasks that met the two criteria above, the degree of misjudgment was calculated. This was done by calculating the vertical distance between the perceived ability measure and the predictions of the linear regression model. The consistency of the degree of misjudgment across tasks was evaluated using a permutation test based on Pearson’s correlation coefficient. Differences in the magnitude of misjudgment due to ageing were evaluated using a Levene’s test for equality of variances. Instead of the common practice of using independent samples t-test for magnitudes, we evaluated variances, because the degree of misjudgment can take on a positive or negative value. In all statistical analyses, p-values below the cut-off value of 0.05 were considered significant.

## Results

**Fig 2:**
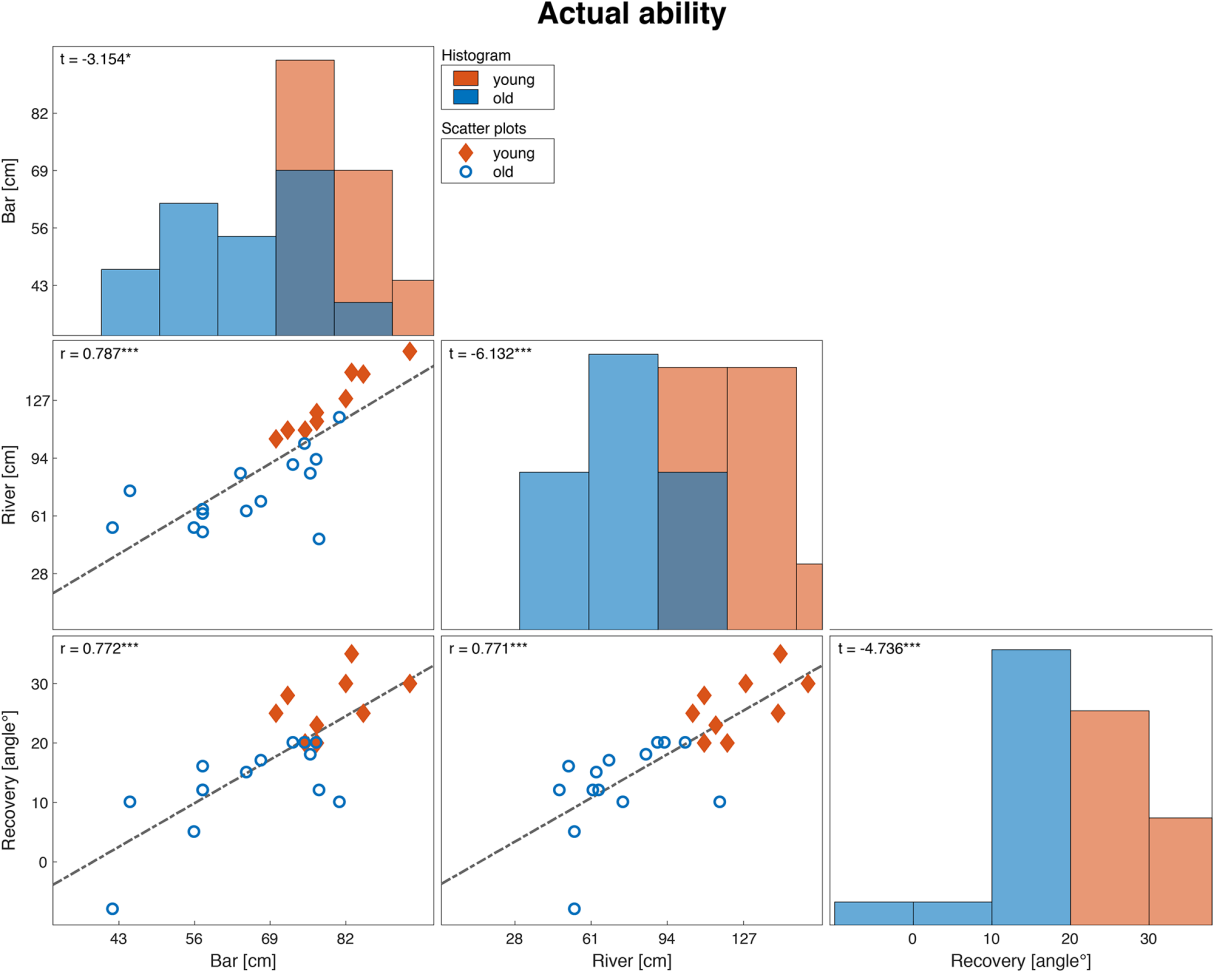
Distribution and correlation matrix of the actual ability measures of the different tasks. The diagonal panels (histograms) show the distribution of the three actual ability measures for the two age groups. The off-diagonal panels (scatter plots) show correlations between actual physical ability measures of different tasks. Diamonds represent young adults and circles represent older adults. Corresponding correlation coefficients and t-tests (i.e., testing the differences between young and older adults) are indicated in the top-left corner of each panel (*:p<0.05, **:p<0.01, ***:p<0.001). Note that the actual ability measure used for the ‘bar’ task was used for the actual ability measure for the ‘cord’ task.

**Fig 3:**
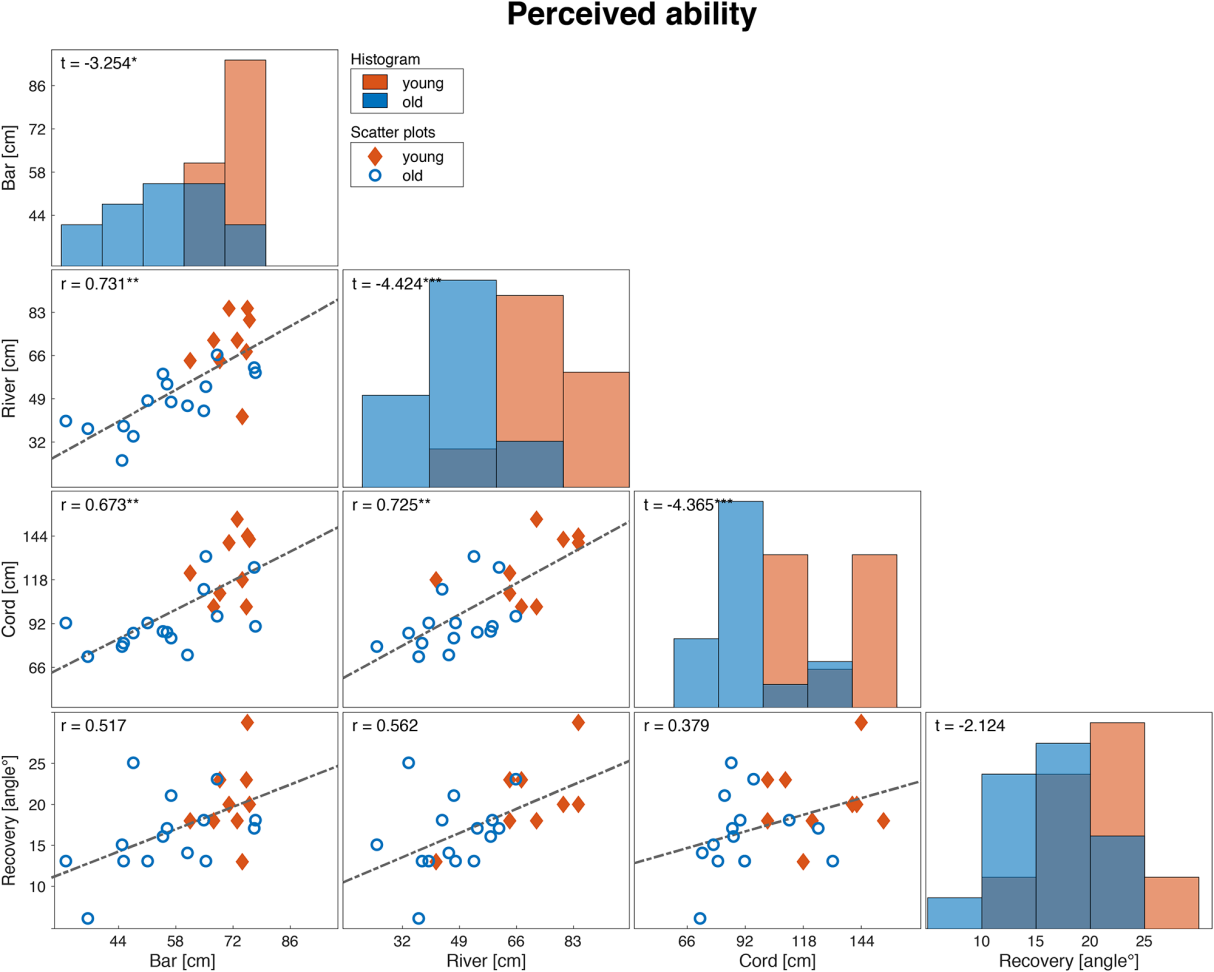
Distribution and correlation matrix of the perceived ability measures of different tasks. The diagonal panels (histograms) show the distribution of the four perceived ability measures of both the age groups. The off-diagonal panels (scatter plots) show correlations between perceived ability measures of different tasks. Diamonds represent young adults and circles represent older adults. Corresponding correlation coefficients and t-tests (i.e., testing the differences between young and older adults) are indicated in the top-left corner of each panel (*:p<0.05, **:p<0.01, ***:p<0.001).

We excluded one participant from the ‘recovery’ task analysis because we could not reliably determine the smallest recovery angle, due to fear of the unexpected release. Overall, young participants had better actual abilities in all stepping tasks than older participants (Fig 2). Similarly, young adults perceived their abilities to be higher than their older peers, except for the perceived ability in the ‘recovery’ task (Fig 3).

Between all tasks, the actual ability measures highly correlated (Fig 2). For the perceived ability measures, all tasks but the ‘recovery’ task were highly positively correlated to all other tasks (Fig 3). These findings suggest that our tasks, except for the ‘recovery’ task, indeed measure valid constructs of perceived and actual physical ability and therefore met our first criteria. Regarding the second criteria of the construct validity, for the ‘bar’ (Δ AIC = 1.86), ‘river’ (Δ AIC = 2.38), and ‘recovery’ task (Δ AIC = 2.62) the linear model appeared to better fit a quadratic alternative. However, in the ‘cord’ task, the alternative model was found to be the more optimal solution (Δ AIC = -1.98).

**Fig 4:**
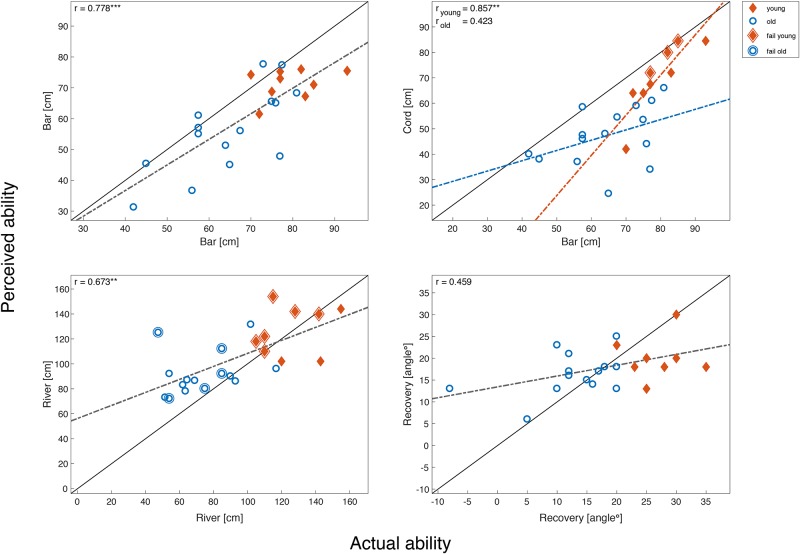
Perceived versus the actual ability for each of the four stepping tasks. A linear regression model was fitted to the data (dotted line). Because of the significant interaction effect with age group in the ‘cord’ task, we plotted the two different linear regression models for each age group. The identity line is indicated by the solid black line. Diamonds are representative for young adults, where circles are indicative for older adults. Participants that failed to successfully step over the string in the ‘cord’ task or step over the ‘river’ are indicated by an extra contour around the diamond or circle. The correlation coefficients are depicted in the top-left corner of each panel (*:p<0.05, **:p<0.01, ***:p<0.001).

All variables, except for the ‘cord’ task, appeared to meet the assumptions of normal distribution, normality of residuals, and homoscedasticity. The actual ability was predictive for the perceived ability in the ‘bar’ and ‘river’ tasks (Fig 4,‘bar’: *r* = 0.778, p<0.001; ‘river’: *r* = 0.673, p = 0.002). No significant correlation between actual and perceived ability was found for the ‘recovery’ task (*r* = 0.459, p = 0.098). A significant interaction effect between age group and actual ability was only found for the ‘cord’ task (*t* = 4.844, p = 0.041).

**Fig 5:**
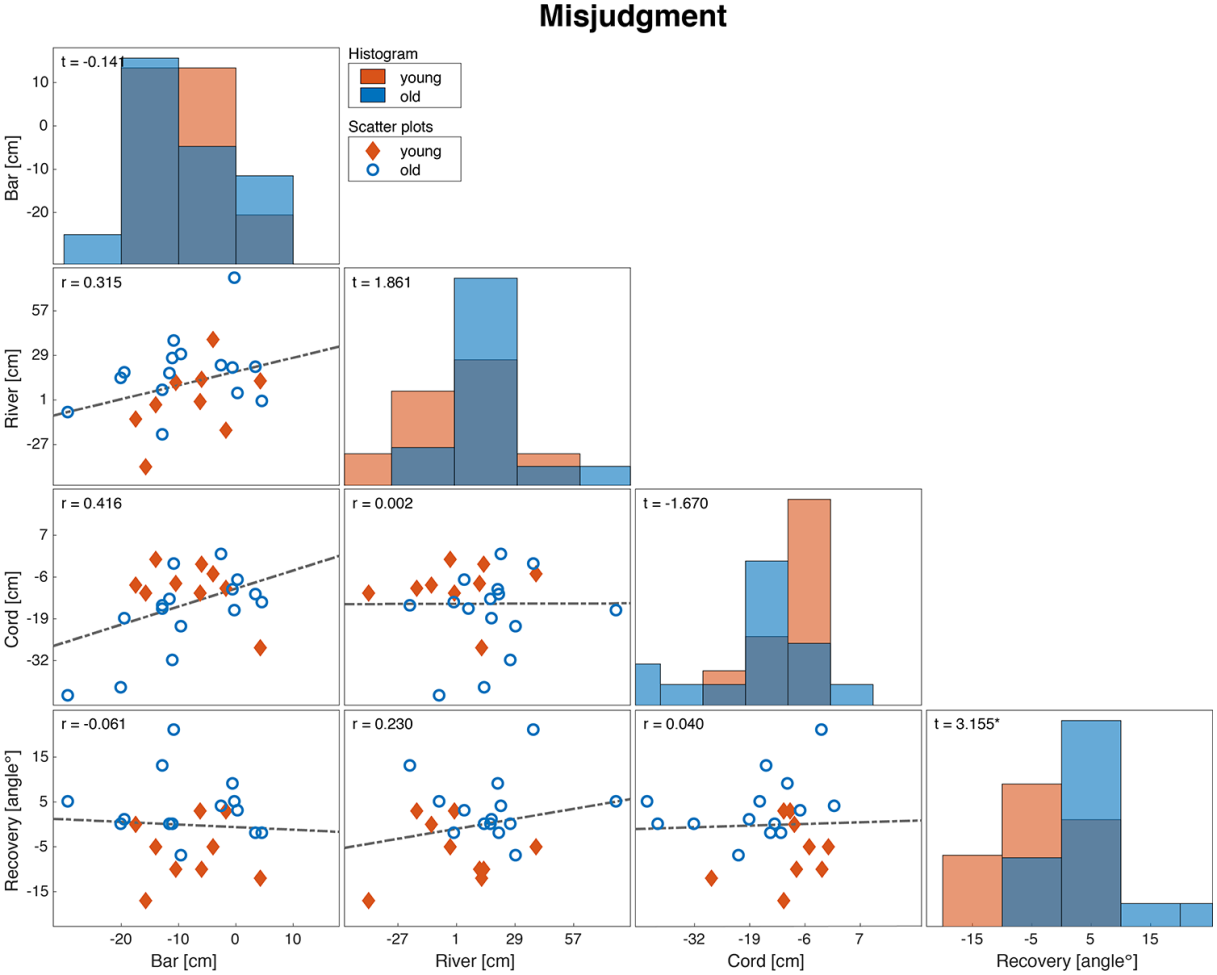
Distribution and consistency matrix of the degree of misjudgment over tasks. The diagonal panels (histograms) show the distribution of the misjudgment for the two age groups, where the off-diagonal panels (scatter plots) show the correlation in the degree of misjudgment between tasks. Diamonds represent young adults and circles represent older adults. Corresponding correlation coefficients and t-tests (i.e., testing the differences between young and older adults) are indicated in the top-left corner of each panel (*:p<0.05, **:p<0.01, ***:p<0.001).

Regarding the consistency, the degree to which participants misjudged their actual ability was not significantly correlated across tasks (Fig 5).

Variances were equal between young and older participants for all tasks (‘bar’:*W* = 0.925, p = 0.347; ‘cord’: *W* = 1.824, p = 0.191; ‘river’: *W* = 0.093, p = 0.763; ‘recovery’: *W* = 0.098, *p* = *0.757*).

## Discussion

The aim of the current study was to assess whether misjudgment transfers across stepping tasks in young and older adults. For this purpose, we proposed criteria to evaluate the construct validity of the different methods for the assessment of the degree of misjudgment. Although we found both perceived abilities as well as actual abilities highly correlated between tasks, we could not find any consistency in the degree of misjudgment between tasks. This might suggest that one’s degree of misjudgment is not an inherent trait, and should be considered as a task-dependent measure. In a study by Rhea and colleagues [32], subjects adjusted their toe elevation after repeatedly stepping over an obstacle, while the obstacle height was perceived similarly over time. It suggests that judgment is updated based on experience, which may make it task-specific and time variant. Yet, some nuances need to be made with respect to our findings. In contrast to previous studies with older adults [13, 17, 18], we found relatively strong associations between perceived and actual ability (Fig 4), implying that overall, our young and older participants were judging their physical abilities quite accurately. Some variability in the degree to which participants misjudge their abilities is required to be able to evaluate its consistency. Having relatively accurate judgers in our sample led to a reduction in variance compared to other studies.

The perceived ability on the ‘recovery’ task was not correlated to perceived ability on any other task, nor was there a relationship between perceived and actual physical ability for the ‘recovery’ task. For all other tasks, the possibility exists that the actual physical ability measure would be affected by the perceived ability, as subjects could make choices in task execution based on perceived ability. For this reason we had included the ‘recovery’ task, because we expected such choices to be limited in this task. Possibly, the ‘recovery’ task induced fear [33] or was too different from what occurs in (voluntary) activity in daily life, which may have complicated making a adequate judgment.

To allow making fair comparisons between tasks, we calculated the degree of misjudgment based on a linear regression model, in contrast to an identity line as described before [17]. However, using a regression model requires that the association does not differ between age groups. Only in the ‘cord’ task we found a significant interaction effect, meaning that the actual physical ability measure had a different relation with the perceived ability in young adults compared to older adults. An explanation could be that perception of risk was different between groups, where older adults were possibly reluctant to cross the line early, while young adults were more confident that they would be able to regain balance in case of an unsuccessful attempt. In support of this explanation, we see that there was no influence of age on the association of the perceived and actual ability measure in the ‘river’ task. In this task, the balance threat is minimal, since failure means stepping on a piece of paper, in contrast to the ‘cord’ task, which contained an actual balance threat.

In contrast with our expectations, misjudgment appeared not to transfer across tasks. This finding could be partly explained by the fact that for two of the four tasks (‘recovery’ and ‘cord’) we could not determine the degree of misjudgment. However, as the other two tasks (‘bar’ and ‘river’) appeared to be valid constructs, we performed an additional analysis to sort out whether other covariates possibly affected our findings. We assumed the slope of the linear regression models for each of our tasks to be similar to the identity line, yet a potential difference between slopes would imply that subjects who performed well were defined as underestimating their abilities, where the ones that performed poorly were accordingly overestimating their ability or vice versa. To check for this assumption, we therefore performed additional t-tests on the regression parameter ${\hat{\beta }}_{1}$ (i.e., testing the null hypothesis that $H_{0}:{\hat{\beta }}_{1}=1$). The slopes of the linear regression models of ‘river’ and ‘recovery’ were significantly different from the slope of the identity line (‘river’: $t_{{\hat{\beta }}_{1}}=-3.934$, p<0.001; ‘recovery’: $t_{{\hat{\beta }}_{1}}=-7.172$, p<0.001). In both the ‘river’ and ‘cord’ task, the perceived ability was assessed by instructing participants to get to the other side as fast as possible, crossing the obstacle at a point which suited the participants. By doing so, participants had the freedom to walk further, benefitting from an easier crossing at the cost of more time spent on the task. Given the slope difference, it can be argued that the benefit-cost ratio for both tasks varies from 1. This means that the benefit of walking further to decrease the width of the crossing does not equal the drawback of increasing the time spent on the task. So despite the valid construct of the ‘river’ task, the degree of misjudgment on this task could not be compared with the ‘bar’ task that did have a similar slope to the identity line.

In addition to the examination of the validation criteria, we compared the misjudgment between young and older adults. No difference between the degree of misjudgment was found, suggesting that older adults are not less accurate judging their ability than their younger peers. In contrast to our findings, Konczak and colleagues [34] did find that young adults were less accurate in estimating their stair climbing ability than older adults, and Sakurai and colleagues [18] found that young adults tended to underestimate their abilities. However, neither of these studies evaluated the validity of their tasks. Our ‘cord’ task did display an interaction effect with age group, which might suggest that older adults used different strategies in indicating their perceived height than young adults. However, due to this interaction, this task no longer allowed us to validly calculate and compare the degree of misjudgment between age groups. Furthermore, our sample might have lacked the power to indicate true differences in the other (valid) tasks. We therefore cannot draw conclusions regarding the possible differences in the degree of misjudgment between age groups and need further studies with valid tasks and larger samples. Note however, that assessing between-group differences was only a secondary aim of this paper, and for the validation analysis of the tests, the subject groups were pooled giving n = 24.

Although we were able to set and check criteria for the validity of the stepping tasks to determine misjudgment, this study had some limitations that need consideration with respect to our aim to unravel whether misjudgment is an inherent trait that transfers to other stepping tasks in young and older adults. First, three out of the four tasks we selected turned out not to be valid for examining the degree of misjudgment. Therefore, to establish a consistency of the degree of misjudgment between stepping tasks, one or more new stepping tasks need to be developed and tested based on the criteria that we have set in this paper. Furthermore, the failure rates on the cord and river tasks were not analysed. In theory, the participants that had chosen a bigger height or distance than that was measured during the actual ability trial should always have failed the trial. This was indeed the case for the ‘cord’. However, a few participants reached higher scores in the perceived ability trial than in the actual ability trial in the ‘river’ task. This might be explained by the use of the approach velocity in stepping over the ‘river’ task, whereas they stood still before their actual maximal forward step. The validity of the ‘river’ task might be improved by giving better instructions on how to cross the river (e.g., first stand still for a moment before stepping, or walk along the river and make a 90 degrees turn before crossing the river).

### Conclusion

The degree of misjudgment of physical ability did not transfer across different stepping tasks. However, only the ‘bar’ and ‘river’ tasks met our criteria for validly assessing the degree of misjudgment, the latter appeared not suitable for comparison across tasks. Based on the finding of the ‘bar’ task only, it remains unclear whether misjudgment of physical ability is task-specific or an inherent trait. Future research on the misjudgment of physical ability should test the construct validity of their methodology by assessing the criteria set in this study.

## Supporting information

S1 File Dataset. (CSV)
